# Smartphone app directs first responders to cardiac arrest three minutes before ambulance

**Published:** 2018

A novel smartphone application (app) has been developed that can direct first responders to cardiac arrest victims more than three minutes before the emergency services arrive. *Each minute increases the chance of survival by 10%.*

The EHRA First Responder app was created by the European Heart Rhythm Association (EHRA), a registered branch of the *European Society of Cardiology (ESC)*. It was released during EHRA EUROPACE – CARDIOSTIM 2017.

‘Sudden cardiac arrest is lethal within minutes if left untreated,’ said EHRA spokesperson Dr Christian Elsner. ‘In Europe, the emergency services arrive around nine minutes after a cardiac arrest. Every minute earlier raises the probability of survival by 10% and reduces the risk of brain injury, which starts four minutes after cardiac arrest.’

If cardiopulmonary resuscitation (CPR) is initiated by a member of the public, this will in essence shorten the time between cardiac arrest and the urgently needed resuscitation measures. However, bystander resuscitation occurs in just of 30–60% of patients who have a cardiac arrest outside hospital.

The EHRA First Responder app was developed to increase the rate of bystander resuscitation and reduce the time between cardiac arrest and resuscitation. Based on GPS tracking technology, the app is used by existing emergency services (reached in many countries by dialling 112) to locate trained ‘app rescuers’ and then automatically direct them to the scene of cardiac arrest. The target is for an app rescuer to arrive three to four minutes after the cardiac arrest.

In a typical scenario, after the cardiac arrest, a bystander calls the emergency services. The operator dispatches an emergency crew and simultaneously locates nearby app rescuers. The nearest app rescuers are notified on their smartphones and the quickest responder is given directions, via the app, to the patient and then performs CPR. Other app rescuers can then additionally bring a nearby automated external defibrillator (AED).

The app was tested in Lubeck, Germany, where around 600 app rescuers were recruited. In 36% of cardiac arrests, an app rescuer arrived more than three minutes before the emergency services. App rescuers were recruited through a local media campaign and 70% were already medically trained. The 30% without medical training took a basic lifesupport course and committed to retaking it every two years.

‘Recruitment of the app rescuers was no problem at all because people want to help,’ said Dr Elsner. Project organisers are now asking emergency dispatch units (fire departments and hospitals) across Germany to connect to the app so that they have free access to the fleet of app rescuers.

‘The software has a standard interface and can be easily connected to most emergency alert systems in Europe in just a few steps,’ said Dr Elsner. ‘We provide insurance for app users and we have a guarantee of data security from the German Department for Data Security in Schleswig– Holstein.

Dr Elsner concluded: ‘Ultimately we will roll the app out across Europe. We hope to raise bystander resuscitation rates to 70–90% and for cardiac arrest patients to be resuscitated in three to four minutes on average.’

For more information, visit: www.firstresponderapp.com
